# Renal Consequences of COVID-19

**DOI:** 10.14797/mdcvj.1058

**Published:** 2021-12-15

**Authors:** Sean A. Hebert

**Affiliations:** 1 Division of Nephrology, Department of Medicine, Houston Methodist, Houston, Texas, US

**Keywords:** COVID-19, SARS-CoV-2, acute kidney injury, kidney transplant, dialysis

## Abstract

The column in this issue is provided by Sean A. Hebert, MD, assistant professor of Clinical Medicine at the Houston Methodist Academic Institute. Dr. Hebert specializes in transplant nephrology at Houston Methodist.

The kidney is one of the major extrapulmonary organs involved in coronavirus disease 2019 (COVID-19) infections, but direct viral access to the kidney is not well understood. Fulminant viremia and viruria are not typical features of COVID-19 patients. Yet, SARS-CoV-2 RNA was detected in kidney tissue in up to 50% of COVID-19 patients on postmortem series.^1^ While our understanding of COVID-19 continues to evolve, consider these important aspects of COVID-19 related to kidney disease.

## Estimates of clinical characteristics in hospitalized COVID-19 patients^2,3^

- Proteinuria: 65.8%–84%
- Hematuria: 41.7%–81%
- Acute kidney injury (AKI): 32%–37%
- Acute kidney injury (AKI) requiring renal replacement therapy (RRT): 12%–15%

## Outcomes^2^

- In-hospital mortality is 50% among patients with AKI compared with 8% in those without AKI.
- Of all patients with AKI, only 30% survived with recovery of kidney function at hospital discharge.

## Hypertension^4^

- There is no evidence that stopping renin angiotensin system inhibitors reduces COVID-19 severity.

## Dialysis^5,6^

- Viral transmission is minimal in outpatient in-center dialysis units due to routine disinfection, personal protective equipment, and cohort isolation.
- Cumulative incidence is 17% to 20% among in-center dialysis patients.
- Increased mortality rate: 25% to 30% compared with the general population of hospitalized COVID-19 patients.

## Kidney transplant^7^

- Increased mortality rate: 13%–30%
- Graft loss: 3.4%–6.3%
- Discontinuing the antimetabolite mycophenolate is recommended for patients with severe infection.

## Vaccine efficacy^8,9,10,11^

- Dialysis:
  - 16% response after first dose compared with 62% in healthy controls
  - 82% response after second dose, but antibody titers are significantly lower than in healthy controls
- Kidney transplant:
  - 15% response after first dose
  - 54% response after second dose, with 46% not developing detectable antibody titers
  - Recipients can receive third dose 28 days or more after completing initial vaccine series
  - 33% response after third dose in patients with no detectable antibodies after second dose

## Treatment

- Administration of bamlanivimab and casirivimab-imdevimab has shown favorable results with minimal adverse effects in solid organ transplant recipients.
- Remdesivir was superior to placebo in shortening time to recovery in hospitalized COVID-19 patients. Importantly, early trials excluded those with acute kidney injury, those on dialysis, or those with kidney transplants. Early studies in these populations have shown encouraging results.

## References

[1] Hassler L, Reyes F, Sparks M, Welling P, Batlle D. Evidence For and Against Direct Kidney Infection by SARS-CoV-2 in Patients with COVID-19. Clin J Am Soc Nephrol. 2021 Jun 14;CJN.04560421. doi: 10.2215/CJN.04560421 PMC8729421 34127485

[2] Chan L, Chaudhary K, Saha A, et al. AKI in Hospitalized Patients with COVID-19. J Am Soc Nephrol. 2021 Jan;32(1):151–160. doi: 10.1681/ASN.2020050615 32883700 PMC7894657

[3] Pei G, Zhang Z, Peng J, et al. Renal Involvement and Early Prognosis in Patients with COVID-19 Pneumonia. J Am Soc Nephrol. 2020 Jun;31(6):1157–1165. doi: 10.1681/ASN.2020030276 32345702 PMC7269350

[4] Lopes RD, Macedo AVS, de Barros ESPGM, et al. Effect of Discontinuing vs Continuing Angiotensin-Converting Enzyme Inhibitors and Angiotensin II Receptor Blockers on Days Alive and Out of the Hospital in Patients Admitted With COVID-19: A Randomized Clinical Trial. JAMA. 2021 Jan 19;325(3):254–264. doi: 10.1001/jama.2020.25864 33464336 PMC7816106

[5] Corbett RW, Blakey S, Nitsch D, et al. Epidemiology of COVID-19 in an Urban Dialysis Center. J Am Soc Nephrol. 2020 Aug;31(8):1815–1823. doi: 10.1681/ASN.2020040534 32561681 PMC7460899

[6] Ng JH, Hirsch JS, Wanchoo R, et al. Outcomes of patients with end-stage kidney disease hospitalized with COVID-19. Kidney Int. 2020 Dec;98(6):1530–1539. doi: 10.1016/j.kint.2020.07.030 32810523 PMC7428720

[7] Azzi Y, Parides M, Alani O, et al. COVID-19 infection in kidney transplant recipients at the epicenter of pandemics. Kidney Int. 2020 Dec;98(6):1559–1567. doi: 10.1016/j.kint.2020.10.004 33069762 PMC7561527

[8] Speer C, Goth D, Benning L, et al. Early Humoral Responses of Hemodialysis Patients after COVID-19 Vaccination with BNT162b2. Clin J Am Soc Nephrol. 2021 Jul;16(7):1073–1082. doi: 10.2215/CJN.03700321 34031181 PMC8425619

[9] Yi SG, Knight RJ, Graviss EA, et al. Kidney Transplant Recipients Rarely Show an Early Antibody Response Following the First COVID-19 Vaccine Administration. Transplantation. 2021 Jul 1;105(7):e72–e73. doi: 10.1097/TP.0000000000003764 33741844

[10] Boyarsky BJ, Werbel WA, Avery RK, et al. Antibody Response to 2-Dose SARS-CoV-2 mRNA Vaccine Series in Solid Organ Transplant Recipients. JAMA. 2021 Jun 1;325(21):2204–2206. doi: 10.1001/jama.2021.7489 33950155 PMC8100911

[11] Werbel WA, Boyarsky BJ, Ou MT, et al. Safety and Immunogenicity of a Third Dose of SARS-CoV-2 Vaccine in Solid Organ Transplant Recipients: A Case Series. Ann Intern Med. 2021 Sep;174(9):1330–1332. doi: 10.7326/L21-0282 34125572 PMC8252023

